# Swiss Cheese Heart: A Tale of Multiple Valve Perforations

**DOI:** 10.14797/mdcvj.1341

**Published:** 2024-04-05

**Authors:** Hussam Al Hennawi, Shayan Iqbal Khan, Aamna Khan, Usama Sadiq, Sung-Hae Cho

**Affiliations:** 1 Jefferson Abington Hospital, Abington, Pennsylvania, US; 2 Thomas Jefferson University Hospital, Philadelphia, Pennsylvania, US

**Keywords:** ventricular septal defect, intracardiac shunting, bicuspid aortic valve, infective endocarditis, Gerbode defect

## Abstract

Gerbode defect, an anomalous connection between the left ventricle and right atrium, is often congenital but can be acquired or iatrogenically formed. We present an exceedingly rare case of this defect associated with multiple valve perforation in an otherwise healthy patient with bicuspid aortic valve and endocarditis.

## Case Presentation

A 31-year-old male with no significant medical history presented with 3 weeks of intermittent fever, fatigue, weight loss, palpitations, and dyspnea. In the emergency department, the patient appeared acutely ill, diaphoretic, and tachypneic. He had a temperature of 101.7°F, blood pressure of 135/47 mm Hg, heart rate of 129 beats per minute, respiratory rate of 36 breaths/minute, and oxygen saturation of 96% on room air. Physical examination was significant for a diastolic murmur at the left lower sternal border with a 3/6 systolic murmur at the apex and water hammer femoral pulse bilaterally.

Laboratory workup was significant for a white blood cell count of 11.4 B/L, troponin of 187 ng/L, NT pro-B natriuretic peptide of 7,800 pg/mL, and a lactate level of 4.7 mmol/L. His chest x-ray showed mild diffuse pulmonary edema and mild cardiomegaly. Electrocardiogram showed sinus tachycardia with first-degree atrioventricular block with poor R wave progression in leads V1-V3. Transthoracic echocardiogram revealed severe aortic regurgitation (AR) with a large irregular mobile mass measuring at least 2 cm. An ejection fraction of 40% to 45% prompted concern for aortic valve (AV) vegetation and left ventricle (LV) dysfunction.

**Figure 1:**
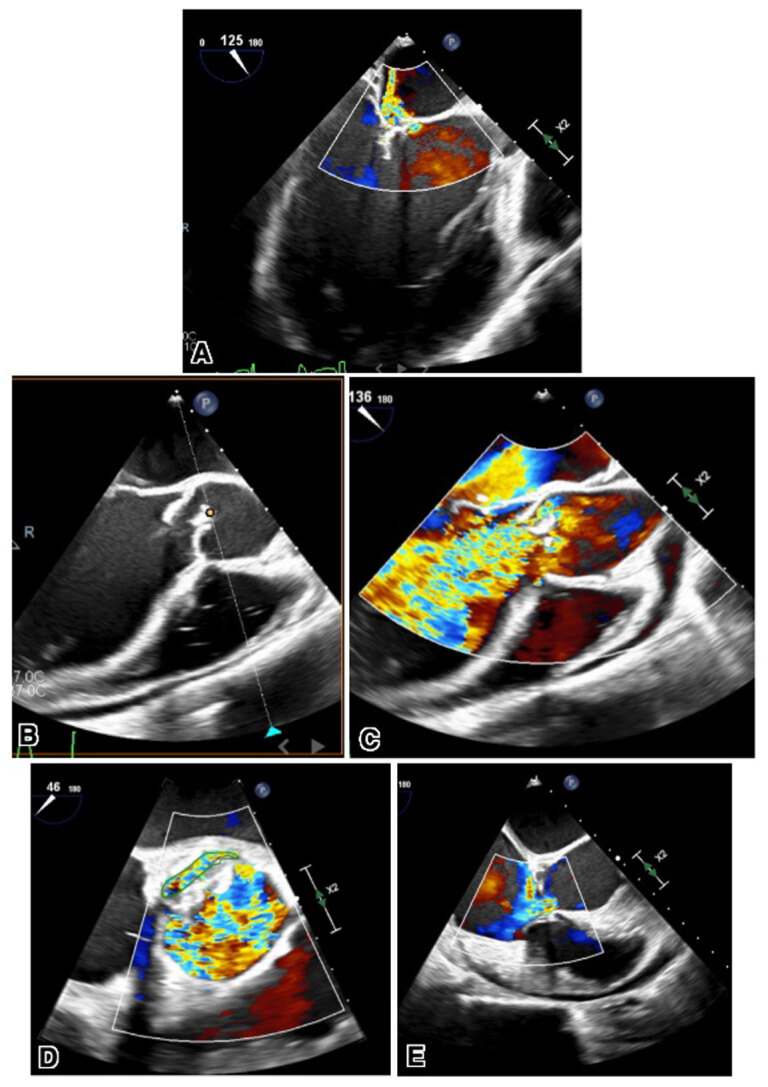
**(A)** Transesophageal echocardiogram images showing perforated anterior leaflet of the mitral valve. **(B)** Long- and short-axis views showing perforated aortic valve **(C, D)** with color flow Doppler. **(E)** Transesophageal echocardiography long-axis view with color flow Doppler showing ventriculoatrial shunt flow from the left ventricle into the right atrium.

Transesophageal echocardiogram further showed bicuspid AV with large vegetation and thickened aortic root concerning for abscess, perforation of the noncoronary cusp of AV with torrential AR, perforation of the anterior leaflet, and severe regurgitation of the mitral valve. Color flow Doppler revealed a large ventriculoatrial shunt with flow originating from the LV and exiting mostly into the right atrium and partially into the right ventricle, consistent with Gerbode defect (Figure 1, Videos 1,2,3,4,5).

**Video 1:** Two-dimensional transesophageal echocardiography long-axis view showing perforated anterior leaflet of the mitral valve; see also at https://youtu.be/pEjZ2OEgRFw.

**Video 2:** Two-dimensional transesophageal echocardiography long-axis view showing perforated aortic valve; see also at https://youtube.com/shorts/JyoJwJF3QJM.

**Video 3:** Two-dimensional transesophageal echocardiography long-axis view with color flow Doppler showing perforated aortic valve; see also at https://youtu.be/cYE_BPoucqE.

**Video 4:** Two-dimensional transesophageal echocardiography short-axis view with color flow Doppler showing perforated aortic valve; see also at https://youtu.be/HrZUch23SbA.

**Video 5:** Two-dimensional transesophageal echocardiography long-axis view with color flow Doppler showing ventriculoatrial shunt flow from the left ventricle into the right atrium; see also at https://youtu.be/LQRjok6eTGM.

The patient was treated with intravenous antibiotics and was moved to the operating room due to his LV dysfunction, magnitude of shunt, concomitant anatomic abnormalities, and hemodynamics, for which he underwent full sternotomy with AV replacement with a bioprosthetic valve and repair of the Gerbode defect with a pericardial patch. The vegetation of the mitral valve anterior leaflet was resected and the anterior leaflet patch was repaired along with the tricuspid valve.

Following the procedure, the patient developed a complete heart block with biventricular failure complicated by cardiogenic shock and polymorphic ventricular tachycardia arrest. He underwent stellate ganglion block and subsequent cardiac resynchronization therapy with a defibrillator. The patient eventually improved, was started on goal-directed medical therapy, and was able to be discharged to his home.

## Discussion

Given the high risk of infective endocarditis (IE) in patients with a bicuspid AV (BAV), particularly when involving IE from the viridans group streptococci that are odontologic in origin, antibiotic prophylaxis to prevent IE should be reconsidered in these patients. The American Heart Association (in 2007 and 2021) and the European Society of Cardiology (in 2009) recommend antibiotic prophylaxis for IE in high-risk patients only and do not recommend antibiotic prophylaxis for IE in BAV patients (who are considered intermediate-risk).^1,2,3^

However, multiple studies now suggest that patients with BAV have clinical risk/profiles that are similar to patients who are at high risk for IE. Hence, antibiotic prophylaxis should be revisited in these patients.^4,5^

## References

[1] Wilson W, Taubert KA, Gewitz M, et al.; American Heart Association Rheumatic Fever, Endocarditis, and Kawasaki Disease Committee; American Heart Association Council on Cardiovascular Disease in the Young; American Heart Association Council on Clinical Cardiology; American Heart Association Council on Cardiovascular Surgery and Anesthesia; Quality of Care and Outcomes Research Interdisciplinary Working Group. Prevention of infective endocarditis: guidelines from the American Heart Association: a guideline from the American Heart Association Rheumatic Fever, Endocarditis, and Kawasaki Disease Committee, Council on Cardiovascular Disease in the Young, and the Council on Clinical Cardiology, Council on Cardiovascular Surgery and Anesthesia, and the Quality of Care and Outcomes Research Interdisciplinary Working Group. Circulation. 2007 Oct 9;116(15):1736-54. doi: 10.1161/CIRCULATIONAHA.106.183095 17446442

[2] Wilson WR, Gewitz M, Lockhart PB, et al.; American Heart Association Young Hearts Rheumatic Fever, Endocarditis and Kawasaki Disease Committee of the Council on Lifelong Congenital Heart Disease and Heart Health in the Young; Council on Cardiovascular and Stroke Nursing; and the Council on Quality of Care and Outcomes Research. Prevention of Viridans Group Streptococcal Infective Endocarditis: A Scientific Statement From the American Heart Association. Circulation. 2021 May 18;143(20):e963-e978. doi: 10.1161/CIR.0000000000000969 33853363

[3] Habib G, Hoen B, Tornos P, et al.; ESC Committee for Practice Guidelines. Guidelines on the prevention, diagnosis, and treatment of infective endocarditis (new version 2009): the Task Force on the Prevention, Diagnosis, and Treatment of Infective Endocarditis of the European Society of Cardiology (ESC). Endorsed by the European Society of Clinical Microbiology and Infectious Diseases (ESCMID) and the International Society of Chemotherapy (ISC) for Infection and Cancer. Eur Heart J. 2009 Oct;30(19):2369-413. doi: 10.1093/eurheartj/ehp285 19713420

[4] Zegri-Reiriz I, de Alarcón A, Muñoz P, et al.; Spanish Collaboration on Endocarditis—Grupo de Apoyo al Manejo de la Endocarditis infecciosa en España (GAMES). Infective Endocarditis in Patients With Bicuspid Aortic Valve or Mitral Valve Prolapse. J Am Coll Cardiol. 2018 Jun 19;71(24):2731-2740. doi: 10.1016/j.jacc.2018.03.534 29903346

[5] Pereira SC, Abrantes AL, António PS, et al. Infective endocarditis risk in patients with bicuspid aortic valve: Systematic review and meta-analysis. Int J Cardiol Heart Vasc. 2023 Jul 28;47:101249. doi: 10.1016/j.ijcha.2023.101249 37547264 PMC10400861

